# Integrating ESG indicators into public health governance as a policy innovation for institutional accountability: a scoping review

**DOI:** 10.3389/phrs.2026.1609720

**Published:** 2026-08-17

**Authors:** Pierluigi Catalfo, Daniele Virgillito, Giuseppe Messina, Caterina Ledda

**Affiliations:** 1 Department of Economics and Business, University of Catania, Catania, Italy; 2 Unit for Finance and Asset Management, Provincial Health Authority of Catania, Catania, Italy; 3 Department of Clinical and Experimental Medicine, University of Catania, Catania, Italy

**Keywords:** corporate social responsibility, ESG indicators, health systems, institutional accountability, multisectoral collaboration

## Abstract

**Objectives:**

To examine how ESG indicators and related frameworks are being translated from corporate disclosure into public health governance.

**Methods:**

Following PRISMA-ScR and the PCC framework, we synthesized 25 studies and grey-literature documents to map definitions, implementation levels, governance uses, barriers, and research gaps. ESG integration was defined as the use of environmental, social, and governance indicators to support accountability, transparency, stakeholder engagement, sustainability alignment, and strategic control in health systems.

**Results:**

ESG-related frameworks may strengthen reporting practices, multisectoral coordination, and alignment with the Sustainable Development Goals when supported by leadership, technical capacity, harmonized indicators, and standardized reporting systems. However, the evidence remains heterogeneous and exploratory. Direct effects on governance performance, health outcomes, or equity are rarely demonstrated. Key gaps include unclear conceptual boundaries between ESG, CSR, SDG monitoring, and sustainability reporting, limited appraisal of reporting versus actual performance, measurement inconsistency, and under-representation of low-resource and Global South contexts.

**Conclusion:**

Future research should validate sector-specific ESG indicators and evaluate whether ESG integration improves accountability, equity, and sustainable public health governance.

## Introduction

Environmental, Social, and Governance (ESG) frameworks have evolved as essential tools for promoting corporate sustainability and responsible business practices. The conceptual foundation of ESG integrates risk management, ethical corporate citizenship, and sustainable value creation [1]. ESG implementation involves various governance dimensions, including organizational settings, strategy, and implementation structures [2]. While developed economies demonstrate more robust ESG integration, emerging markets face challenges such as fragmented governance and limited institutional capacities [3]. ESG practices are increasingly incorporated into corporate governance frameworks, influencing board composition, executive compensation, and transparency practices [4]. The theoretical underpinnings of ESG research include sustainability of competitive advantage, compliance of social construction, and alignment of governance accountability [5]. Various ESG frameworks, such as GRI, PRI, and SASB, offer different approaches to sustainability reporting and implementation [6].

To avoid treating ESG as a generic synonym for sustainability, this review distinguishes between ESG as a corporate disclosure framework and ESG as a public governance instrument. In its original corporate use, ESG primarily organizes non-financial information on environmental risks, social responsibilities, and governance practices for investors, regulators, and stakeholders. In public health governance, ESG integration is instead understood as the deliberate incorporation of those indicators into stewardship, priority setting, planning, monitoring, reporting, and accountability processes.

This distinction guided the eligibility criteria. Studies on SDGs, CSR, sustainability metrics, and governance indicators were included only when they contributed to at least one ESG-relevant governance function: transparency, accountability, participation, equity, institutional control, risk management, or sustainability alignment. Thus, ESG-related evidence was mapped as an emerging governance architecture rather than as a single standardized measurement system.

The literature highlights significant gaps in public health governance, particularly in transparency, accountability, and strategic control mechanisms. Key challenges include weak policy implementation, limited cross-sector coordination, and inadequate understanding of accountability frameworks [7]. Corruption and lack of transparency in developing countries’ health services are major obstacles to effective service delivery [8]. The governance accountability problem (GAP) reveals a disconnect between *ad hoc* interventions and state responsibilities for health security [9]. Public-private partnerships in global health require improved transparency and accountability for better performance [10]. In rural settings, strong accountability towards management and partners often fails to extend to the public [11]. Enhancing citizen participation, improving human resource capacity, and adopting technology-based systems are proposed strategies to strengthen governance and accountability in public health [7, 12, 13].

The application of ESG principles to the public health sector is gaining traction as a strategic evolution of value-based healthcare [14]. ESG implementation in healthcare can contribute to sustainability goals, improve financial performance, and enhance risk management [15, 16]. While the healthcare industry has traditionally focused on social aspects, there is a growing emphasis on environmental considerations due to the sector’s significant carbon footprint [15]. ESG principles can serve as a framework for non-financial reporting in healthcare organizations, although adaptation to specific organizational missions is crucial [17]. The integration of ESG in healthcare aligns with the UN Sustainable Development Goals, particularly SDG 3 on health and wellbeing [18]. However, challenges remain in implementing ESG principles, especially in emerging markets and nonprofit organizations [16, 17].

The integration of ESG principles into public health governance and risk management is gaining strategic importance globally. ESG integration enhances governance quality, stakeholder trust, and institutional resilience [3]. It is crucial for achieving sustainability goals and offers potential economic benefits in healthcare [15]. ESG adoption improves risk management, operational efficiency, and corporate reputation [19]. Adding health considerations to ESG criteria (ESG + H) could catalyze the inclusion of health in financial decision-making [20]. An integrated population health risk management framework can facilitate evidence-based health policy development [21]. However, challenges remain, including inadequate tools and resources, particularly in emerging economies [22]. Effective ESG integration requires aligning with strategic planning, establishing robust governance frameworks, and fostering a risk-aware culture [23, 24].

The concept of good governance in health systems has evolved over time, encompassing principles such as accountability, transparency, participation, and effectiveness [25, 26]. It emerged from different disciplines, including economics, political science, and development studies [27]. Good governance is seen as crucial for improving health system performance and achieving better health outcomes [28]. Key milestones include the shift from social protection to health policy and governance paradigms [29], and the recognition of governance’s role in achieving health-related Millennium Development Goals [30]. Challenges in implementing good governance include measuring outcomes, adapting to local contexts, and bridging the gap between policy and practice [31, 32]. Various frameworks have been developed to assess health system governance, though only a few have been applied in practice [27].

Governance in public sector health systems involves multiple dimensions, with transparency and accountability emerging as key principles [10, 27]. These concepts are often interconnected and contribute to improved organizational performance and public trust [33]. Various frameworks have been developed to assess health system governance, drawing from disciplines such as economics, political science, and public management [27]. The TAPIC framework, for instance, identifies five domains: Transparency, Accountability, Participation, Integrity, and Capacity [34]. Challenges in implementing effective governance include weak policy execution, limited cross-sector coordination, and inadequate understanding of accountability frameworks [7, 35]. Strategies for improvement include digitalization of processes, enhancing community participation, and adopting complementary accountability approaches [7, 11]. Research in this field has increased, particularly since the rise of public sector governance as a focal point [36].

Recent research on ESG indicators in healthcare highlights their growing importance for sustainability and financial performance. Studies show that ESG implementation in healthcare management is crucial for achieving Sustainable Development Goals [15]. However, there’s a lack of consensus on defining sustainability and its impact on organizational performance across the three ESG pillars [37]. The relationship between ESG activities and firm performance varies between developed and developing economies [38]. Research gaps exist, particularly in social and governance measures [39]. The healthcare sector’s significant environmental impact necessitates urgent research to inform policy and practice [40]. Future research should focus on developing standardized sustainability metrics, revising infection control standards, and increasing federal funding for sustainability research in healthcare [18, 41].

International governance frameworks for transparency, accountability, and sustainability in health systems and public institutions include the World Health Organization (WHO), Global Reporting Initiative (GRI), and frameworks developed by the World Bank and United Nations Development Programme (UNDP) [42–44]. These frameworks emphasize principles such as strategic vision, participation, rule of law, transparency, responsiveness, equity, effectiveness, accountability, and ethics [42]. The GRI framework has been applied to assess sustainability in healthcare organizations globally [45]. Governance in global health operates across three spaces: global health governance, global governance for health, and governance for global health [12]. Transparency and accountability are crucial for assessing and designing governance in global health partnerships [10]. Anti-corruption measures, including community monitoring and fraud control, are essential for strengthening health systems and achieving universal health coverage [46].

Recent studies highlight the growing adoption of ESG principles in public governance domains outside healthcare. In education, universities are implementing ESG practices to promote sustainability and social inclusion [47, 48]. The integration of ESG principles into environmental education is being explored in the MENA region to address pressing environmental challenges [49]. In public administration, ESG considerations are enhancing government transparency, reducing debt, and attracting foreign investment [50]. The interplay between public and corporate governance in driving ESG engagement has been examined across multiple countries [51]. Some researchers argue for applying ESG principles to both public and corporate governance. In Brazil, the potential application of ESG principles in public administration is being analyzed [52]. These studies demonstrate the increasing relevance of ESG principles in various public governance domains.

## Methods

This scoping review aims to explore the extent and nature of evidence on the integration of ESG indicators into public health governance, as a policy innovation for institutional accountability and control. Following the methodological framework proposed by Arksey and O’Malley [53] and refined by Levac et al. [54], and reported according to PRISMA-ScR guidelines [55].

A scoping review was selected because the evidence base is still emerging, conceptually heterogeneous, and composed of policy documents, conceptual papers, reporting studies, and empirical evaluations. The objective was therefore to map the range of frameworks, definitions, governance applications, implementation conditions, and knowledge gaps, rather than to estimate a pooled effect or determine intervention effectiveness. Consistent with scoping review methodology, a formal risk-of-bias assessment was not conducted; instead, the synthesis explicitly distinguishes conceptual claims, implementation experiences, reporting practices, and empirical outcome evidence.

In order to ensure methodological transparency and to precisely delineate the scope of the review, the inclusion and exclusion criteria were structured according to the PCC framework (Population, Concept, Context) recommended by the Joanna Briggs Institute for scoping reviews [56, 57]. This approach allowed us to systematically define the relevant population (e.g., public health institutions, health systems, and regulatory bodies), the core concept under investigation (i.e., the integration of ESG indicators, sustainability metrics, SDGs, or corporate social responsibility frameworks), and the context (e.g., public health governance, institutional accountability, and national or international policy settings) (Table 1).

**TABLE 1:** PCC framework and eligibility criteria. Source: compiled by the authors from the scoping review synthesis (2026).

PCC element	Definition	Application to this review	Inclusion criteria	Exclusion criteria
Population	The target subjects, organizations, or entities of the investigation	Public health institutions, health systems, regulatory bodies, governmental or intergovernmental organizations involved in public health governance	Studies, reports, or documents focusing on public institutions or health systems at national or international levels	Studies exclusively focused on the private sector or single companies with no direct connection to public health governance
Concept	The central phenomenon or topic of interest	Integration of environmental, social, and governance (ESG) indicators, sustainability metrics, SDGs, or corporate social responsibility (CSR) frameworks	Studies describing or assessing the use, adoption, or impact of ESG indicators, SDGs, CSR, or sustainability metrics in policies or governance frameworks	Studies not addressing or not directly linking ESG indicators, SDGs, CSR, or sustainability metrics to public health governance
Context	The setting or environment in which the concept is applied	Public health governance, institutional accountability, control, and policy innovation at national or international levels	Studies and documents addressing public health governance or institutional accountability mechanisms	Studies not related to public health governance or exclusively addressing clinical aspects or internal management without a broader public governance perspective

Operationally, ESG integration was defined as the use of environmental, social, and governance indicators or ESG-related reporting logics within public health decision-making, monitoring, accountability, or institutional control. Broader SDG, CSR, or sustainability studies were included only when they addressed these functions in public health institutions, health systems, or policy settings.

### Information sources and search strategy

A comprehensive search strategy was developed in consultation with an experienced research librarian to ensure the identification of all relevant sources of evidence. We searched the following electronic databases: PubMed/MEDLINE, Scopus, Web of Science Core Collection, Embase, and CINAHL, from inception to 29 July 2025. In order to capture additional evidence beyond peer-reviewed literature, we also searched relevant grey literature sources, including WHO Institutional Repository, OECD iLibrary, World Bank Open Knowledge Repository, UN Sustainable Development Goals Knowledge Platform, and Google Scholar (first 200 results). Reference lists of all included studies and relevant reviews were hand-searched to identify additional eligible documents.

The search strategy is provided in Supplementary Table S1.

To streamline the elimination of duplicates and facilitate the management of the records identified for inclusion, Rayyan software was used [58]. Three independent reviewers (P.C., D.V., and C.L.) were involved in the study selection process. Screening was performed in two sequential phases. In the first phase, two reviewers (P.C. and D.V.) independently examined the titles, abstracts, and keywords of all retrieved records to assess their alignment with the scope of the review. Records not meeting the inclusion criteria were excluded at this stage. Relevant review articles were also identified for citation tracking in order to uncover additional studies not indexed in the selected databases [59]. In the second phase, the same reviewers independently assessed the full-text articles against the predefined inclusion and exclusion criteria. Disagreements between reviewers were resolved by discussion and, when necessary, by consultation with the third reviewer (C.L.), who supervised the process and ensured methodological consistency across both phases. The entire selection process was documented using a PRISMA-ScR flow diagram.

Extracted information included study type, geographical scope, institutional level, ESG-related framework, ESG dimension, implementation level, accountability mechanism, reported governance outcome, enablers, barriers, and limitations. The synthesis was conducted in two steps. First, evidence was mapped descriptively according to the PCC framework. Second, an analytical matrix was developed to identify recurring ESG dimensions, governance functions, and evidentiary limitations across studies (Table 2). This approach was used to avoid inferring causal effects from studies that primarily provided conceptual, descriptive, or reporting-based evidence.

**TABLE 2:** Analytical synthesis of ESG dimensions, governance uses, and evidentiary limitations. Source: compiled by the authors from the scoping review synthesis (2026).

ESG dimension	Examples of mapped indicators or framework elements	Governance use in public health	Evidence pattern	Main limitation
Environmental	Emissions, resource consumption, waste, green procurement, environmental criteria in health technology assessment, sustainability reporting	Supports monitoring of the environmental footprint of health services and alignment with sustainability commitments	Reported mainly in organizational healthcare studies, procurement policies, and environmental sustainability assessments	Indicators are not consistently standardized and are often weakly connected to health-system accountability outcomes
Social	Equity, social determinants of health, access, community engagement, stakeholder participation, workforce responsibility, patient and population wellbeing	Links ESG to inclusion, responsiveness, participation, and fair distribution of health-system benefits and burdens	Present in SDG, CSR, and urban or community governance studies, but less consistently operationalized than environmental and governance domains	Social indicators remain underdeveloped and are rarely evaluated through longitudinal or intervention designs
Governance	Transparency, disclosure quality, leadership, compliance, anti-corruption, accountability mechanisms, regulatory alignment, board or institutional oversight	Strengthens institutional control, reporting, strategic planning, and accountability to regulators, communities, and stakeholders	Frequently reported across policy, health-system governance, disclosure, and organizational ESG literature	Evidence often demonstrates reporting or procedural change rather than direct improvements in governance performance or health outcomes
Cross-cutting implementation	Standardized reporting systems, data infrastructure, technical capacity, multisectoral collaboration, legal mandates, and local adaptation	Determines whether ESG moves from disclosure to actionable governance and policy learning	Common enablers and barriers recur across settings, especially data gaps, regulatory fragmentation, and limited capacity	Generalizability is limited by heterogeneous study designs, high-income-country concentration, and scarce evidence from low-resource settings

## Results

### Literature search

**FIGURE 1:**
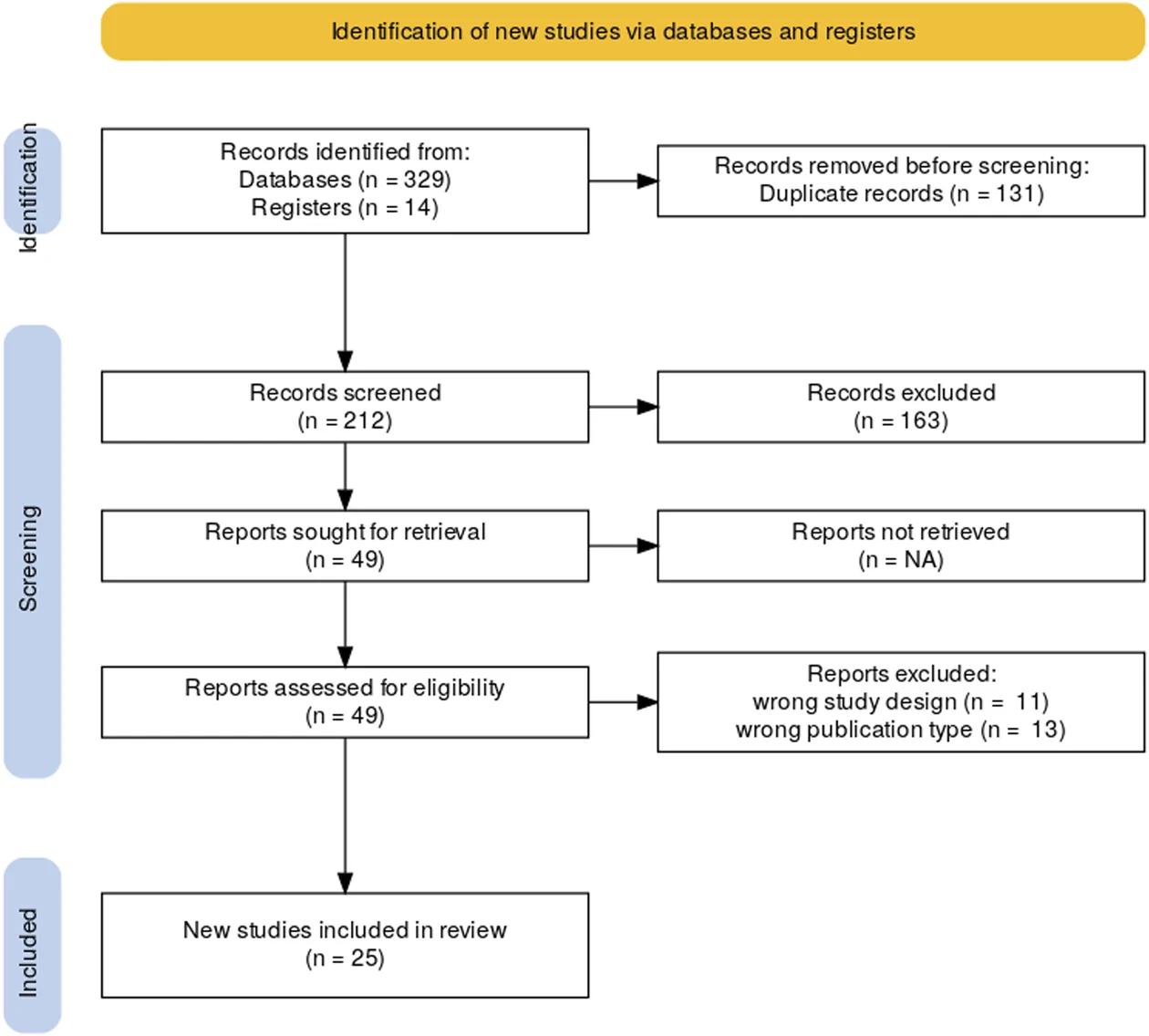
PRISMA-ScR flow diagram of the study selection process for the scoping review. The review followed the methodological framework of Arksey and O’Malley, as refined by Levac et al., and was reported according to PRISMA-ScR guidelines; database searches (PubMed/MEDLINE, Scopus, Web of Science Core Collection, Embase, and CINAHL, plus grey-literature sources) were executed from inception to 29 July 2025.

A comprehensive search of multiple electronic databases initially identified 343 records. The PRISMA flowchart (Figure 1) illustrates the stepwise selection of relevant publications throughout the review process. Following the removal of 131 duplicate records, 212 records remained for the screening stage. During this phase, 163 records were excluded for not meeting the predefined inclusion criteria. Consequently, 49 articles were retrieved for full-text eligibility assessment. In the final stage, 25 studies were included in the qualitative and quantitative synthesis.

### General characteristics of the included studies

A total of 25 studies were included in this review, encompassing a broad range of methodological approaches, geographical scopes, and frameworks related to the integration of ESG indicators into public health governance (Table 3).

**TABLE 3:** General characteristics of the studies included in the scoping review. Source: compiled by the authors from the scoping review synthesis (2026).

Author and Year	Study type	Geographic scope	ESG framework type	Primary focus area	Frameworks/Indicators	Implelemtation level	Accountability measure	Key outcomes	Success factors	Barriers	Institutional chances	Sustainability indicators
Working group for monitoring action on the social determinants of health., 2018 [60]	Policy analysis	Global, 12 countries	Social determinants of health (SDH) action indicators	Monitoring SDH and health equity	Social determinants of health (SDH) action indicators	Global	Indicator selection	Data/indicator gaps, focus on equity	Core indicators, equity focus	Data gaps	Recommend more indicators	SDH action indicators
Al Amosh and Khatib, 2025 [61]	Quantitative	Europe (10 countries, 726 companies)	GRI, ESG	COVID-19, ESG, and sustainability reporting	GRI, ESG	Regional	ESG scores, GRI compliance	GRI equals resilience, non-GRI equals negative	GRI compliance	Non-compliance	Recommend GRI adoption	ESG, sustainability scores
Bennett et al., 2020 [62]	Mixed-methods	Global (policymakers)	SDGs, health policy and systems research (HPSR)	SDGs and health policy research priorities	SDGs, health policy and systems research (HPSR)	Global	Interviews, reviews	Participatory/accountable institutions	Multidisciplinary collaboration	Sustainability of nongovernmental organizations	Recommend long-term view	Accountability measures
Bickler et al., 2020 [63]	Policy analysis; semi-quantitative analysis	World health organization (WHO) european region (20 countries)	WHO europe sustainable development goals (SDG) roadmap	Integration of sustainable development goals (SDGs) in national health governance	WHO europe SDG roadmap	National	Voluntary national review (VNR) assessment, 41 criteria	Good governance, weak social integration	Good governance, multipartner cooperation	Weak social integration	Use of SDG roadmap	Governance, monitoring
Bobini and cicchetti, 2024 [64]	Qualitative study	20 countries (HTA organizations)	Environmental sustainability in HTA	Environmental sustainability in health technology assessment	Environmental sustainability in HTA	Global	Survey, desk analysis	Few organizations active, data/interdisciplinary gaps	Interdisciplinary approach	Data, engagement gaps	Recommend collaboration	Environmental sustainability in HTA
Bosco et al., 2024a [65]	Conceptual/theoretical review	Italy	ESG processing map	ESG strategy in healthcare organizations	ESG processing map	Organizational (healthcare)	Map-based assessment	Framework for ESG strategy	ESG processing map	Regulatory gaps	Map guides strategy	ESG assessment areas
Bosco et al., 2024b [66]	Conceptual/theoretical review	Not specified in abstract	ESG process map	ESG in public knowledge-intensive organizations	ESG process map	Organizational (public knowledge-intensive organizations)	Social network analysis (SNA) alignment	Lack of strategic interest	Social network analysis alignment	Lack of interest	ESG process map	ESG themes
Brizolla et al., 2024 [67]	Qualitative study	Not specified (3 health cooperatives)	ESG, compliance	Compliance and ESG in health cooperatives	ESG, compliance	Organizational (cooperatives)	Compliance programs	Effective governance, transparency	Compliance programs	No mention found	Strengthened governance	ESG actions, transparency
Capobianco et al., 2022 [68]	Policy analysis	Europe	ESG in procurement	Sustainability in healthcare procurement	ESG in procurement	Europe	Policy analysis	No mention found	No mention found	No mention found	No mention found	No mention found
Dossa, 2025 [69]	Quantitative (panel data)	China	ESG disclosure	ESG and financial performance in health firms	ESG discosure	National	Panel data	ESG boosts financial performance, environmental trade-offs	ESG boosts financial performance	Environmental trade-offs	Recommend tailored strategies	ESG disclosure score
Frazer et al. 2021 [70]	Quasi - experimental	Senegal (110 communes)	Integrated governance	Governance and health service delivery	Integrated governance	Local (Senegal)	Quasi-experimental	Improved access, quality	Integrated governance	Siloed funding	Improved access/quality	Service readiness
Fusheini et al., [71]	Case study	South Africa (1 hospital)	Good governance principles	Governance in public hospitals	Good governance	Institutional	Case study	Mixed governance, external constraints	Good governance	External constraints	Recommend improvements	Governance principles
Hone et al., 2017 [72]	Observational, regression	Brazil (1,622 municipalities)	Health governance indicators	Governance and amenable mortality	Health governance	Municipal	Governance scores	Lower mortality with strong governance	Strong governance	Weak oversight, accountability	Recommend benchmarking	Amenable mortality
Jain et al., 2023 [73]	Mixed-methods	United States (14 health plans)	Corporate social responsibility (CSR), social determinants of health (SDOH)	CSR for social determinants of health	Corporate social responsibility (CSR), social determinants of health (SDOH)	National	CSR integration	SDOH as competitive advantage	Social determinants of health integration	“Wrong pocket” problem	CSR as competitive advantage	Social determinants of health, CSR metrics
Kehinde et al., 2023 [74]	Ex-post facto, regression	Sub-saharan Africa (15 companies)	ESG disclosure	Value relevance of ESG in public health	ESG disclosure	National	Regression, variance inflation factor (VIF)	ESG greater than financial performance for value, social weak	Governance disclosure	Social disclosure weak	Recommend compliance systems	ESG versus financial performance value
Martins and paes-sousa, 2024 [75]	Qualitative study	Global (195 voluntary national reviews)	SDG health indicators	Accountability for health-related SDGs	SDG indicators	National	VNR indicator analysis	Paradox: Capacity vs. governance	Technical capacity	Proxy indicators, politics	Recommend standardization	Health SDG indicators
Moldovan and Blaga, 2022 [76]	Qualitative study	Not specified in abstract	San-Q framework	Organizational governance indicators in healthcare	San-Q framework	Organizational (hospital)	8 governance indicators	Validated indicators for governance	Validated indicators	No mention found	Indicator-based assessment	Governance indicators
Paridhi et al., 2024 [77]	Quantitative (panel data)	India	ESG reporting	ESG and financial performance in healthcare	ESG reporting	National	Panel data analysis	ESG lags, environmental/governance pillars key	ESG pillars	COVID-19 impact	Recommend actionable steps	ESG-financial performance relationship
Pizzi et al., [77]	Qualitative study	Italy (202 entities)	SDG3, social reporting	Accountability and transparency in SDG3 reporting	SDG3, social reporting	National	Social report presence	Low accountability, asymmetric information	Social reporting	Low adoption, information asymmetry	Recommend non-financial tools	Social report presence
Pratici et al., 2024 [17]	Qualitative case study	Italy (4 nonprofit organizations)	ESG paradigm	ESG in nonprofit healthcare social reporting	ESG paradigm	Organizational (nonprofit organizations)	Social reporting, interviews	ESG as guide, risk of formalism	ESG as guide, customization	Formalism risk	Tailored ESG use	Social reporting
Roschnik et al., 2019 [79]	Policy analysis	England	Sustainable development unit, carbon reporting	Embedding sustainability in national health service (NHS) governance	Sustainable development unit	National	Carbon reporting, board plans	Carbon reduction, stakeholder engagement	Stakeholder engagement	No mention found	Carbon reduction, reporting	Carbon, energy savings
Scott et al., 2018 [80]	Mixed-methods	Global (policymakers)	Accountability, SDGs	Participatory/accountable institutions	Accountability, SDGs	Global	Interviews, reviews	Political factors, system-level change	Political, system-level	Context, actor influence	Recommend policy integration	Accountability mechanisms
Wójtowicz and Wójtowicz, 2024 [81]	Qualitative study	European Union (50 hospitals)	ESG disclosure index	ESG disclosure practices in public hospitals	ESG disclosure index	Organizational (hospitals)	Website disclosure	Fragmented, governance focus	Governance disclosure	Lack of standards	Recommend standard tool	ESG disclosure index
Wu, 2024 [82]	Mixed-methods	Global, south America, Asia	ESG (Johnson & Johnson case)	ESG in pharmaceutical industry	ESG (Johnson & Johnson)	Global	ESG reporting	Impact on equity, emissions, wellbeing	Accountability, leadership	ESG infancy	Recommend critical review	Emissions, equity, wellbeing
Zariņš and siders, 2025 [83]	Policy analysis, mixed-methods	Latvia, France, Germany	Corporate sustainability reporting directive (CSRD), european sustainability reporting standards (ESRS)	Legal perspectives on sustainability reporting	Corporate sustainability reporting directive (CSRD), european sustainability reporting standards (ESRS)	National	Legal analysis	Double materiality, legal gaps	Double materiality	Legal gaps	Recommend legal reforms	CSRD/ESRS compliance

Given the broad inclusion criteria, the evidence base should be interpreted as a heterogeneous map rather than a set of directly comparable effectiveness studies. Studies differed by institutional level, theoretical foundation, indicator set, and outcome definition. Healthcare organizations and corporate healthcare actors tended to emphasize disclosure, environmental performance, and financial or reputational outcomes, whereas public health governance and health-system studies more often focused on accountability, equity, community participation, and policy alignment.

Policy analyses [60, 63, 68, 75, 83, 84] examined the role of national and regional governance mechanisms and voluntary reporting in driving institutional accountability. Other study [62, 85–89] provided evidence on how integrated governance frameworks, SDG monitoring, and participatory mechanisms contribute to improved governance outcomes across health systems.

Several qualitative studies [17, 64, 67, 90] explored ESG and CSR applications at the organizational and cross-sectoral level, with a focus on stakeholder engagement and social responsibility in healthcare settings. Quantitative and mixed-methods studies [61, 69, 70, 72–74, 82] investigated ESG adoption trends, reporting practices, and associations with institutional performance indicators such as financial stability, mortality reduction, and service delivery outcomes. Conceptual and theoretical contributions [65, 66, 78] highlighted the importance of structured ESG processing maps, disclosure frameworks, and integrated sustainability strategies to strengthen institutional accountability [17, 60, 62, 76–83].

Geographically, 11 studies had a global or multi-country focus, while 9 studies focused on Europe or the United Kingdom. Four studies examined African contexts, and four were based in Asia. Only one study each addressed the United States and Brazil, while three others did not specify a location.

The frameworks and approaches adopted were heterogeneous. ESG-related instruments such as disclosure indexes, ESG paradigms, and processing maps were used in 12 studies [17, 61, 65–67, 69, 74, 78, 82]. SDG-based frameworks, including the WHO Europe SDG Roadmap and SDH action indicators, were reported in seven studies [60, 62, 63, 87–89]. Other approaches included governance indicators or mechanisms [84–86], CSR frameworks [73, 90], environmental sustainability indicators in HTA [64], and international reporting standards such as GRI, SASB, and TCFD [61, 82].

The majority of studies analyzed governance and accountability as their primary focus, including [60, 62, 63, 68, 70, 72, 84–88], while others concentrated on sustainability [64–66, 78], reporting and disclosure practices [17, 61, 69, 74, 82], and health outcomes or equity issues [72, 73, 89].

Across the included studies, recurrent enablers of ESG integration included robust governance structures, stakeholder engagement, and multisectoral collaboration. For instance, Bickler et al. [63] and Martins and Paes-Sousa [75] emphasized the relevance of standardized indicators in facilitating Voluntary National Reviews and improving institutional accountability. Conversely, data gaps [61, 82], inconsistent standards [69, 74, 82], limited resources, and regulatory weaknesses [74, 83] were commonly cited barriers. These challenges often limited the capacity of ESG frameworks to translate policy ambitions into measurable improvements in public health governance and outcomes.

Across the mapped evidence, environmental indicators were most often connected to sustainability reporting, procurement, emissions, resource use, and environmental criteria in health technology assessment. Social indicators were linked to equity, social determinants of health, community engagement, stakeholder participation, and workforce or patient-related responsibility. Governance indicators were linked to transparency, disclosure quality, compliance, leadership, institutional control, anti-corruption, and accountability mechanisms. However, the strength of evidence varied: most studies described frameworks, reporting practices, or implementation experiences, while few tested whether ESG integration produced measurable improvements in governance performance or health outcomes.

### Results of characteristics referred to PCC framework

The 25 studies included in this review (Table 3) analyzed diverse populations, concepts, and contexts in relation to the integration of ESG indicators into public health governance. In terms of population, most studies [60, 63, 68, 75, 83, 84] focused on public health governance structures at the national or regional level, including governmental agencies, health ministries, and public health institutions. Others concentrated on organizational settings such as hospitals, health technology assessment bodies, and healthcare networks [17, 64–67]. Broader health systems and urban governance dynamics were addressed in studies [85, 88, 89], while other contributions examined populations indirectly through corporate, financial, or community health perspectives [69, 73, 74, 82, 90].

With respect to the concept, the studies examined a wide range of frameworks used to integrate ESG indicators, sustainability metrics, or related tools to reinforce institutional accountability. ESG-related frameworks such as disclosure indexes, paradigms, and processing maps were highlighted in several studies [17, 61, 65, 66, 69, 74, 78, 82]. Other studies focused on SDG-based frameworks, including the WHO Europe SDG Roadmap and SDH action indicators [60,62,63,87–89], governance indicator sets [84–86], CSR frameworks [73, 90], and environmental sustainability indicators, particularly in health technology assessment [64]. These frameworks were primarily applied for non-financial reporting, indicator mapping, and policy evaluation, and were often explicitly linked to institutional accountability outcomes.

The context of the studies was equally diverse. Eleven studies adopted a global or multinational scope [60, 62, 85, 87–89], whereas nine focused on European or UK settings [61, 63, 65, 66, 68, 83, 84]. African contexts were addressed in four studies [67, 74, 90], while another four were set in Asia [61, 73, 82]. Only one study each focused on the United States [73] and Brazil [72].

Overall, the findings across the three PCC domains indicate that ESG and related sustainability frameworks are increasingly being applied in varied health governance contexts and at different levels of institutional complexity. Studies consistently reported that effective integration of ESG indicators was facilitated by strong governance structures, standardized reporting frameworks, and multipartner collaboration.

## Discussion

This scoping review synthesized evidence from 25 studies examining how ESG indicators and related frameworks are being integrated into public health governance. The results show that ESG frameworks are increasingly viewed as mechanisms for strengthening institutional accountability, promoting transparency, and aligning public health systems with global sustainability goals. However, the findings also highlight significant heterogeneity in approaches, levels of implementation, and reported outcomes.

These findings should therefore be read as evidence of the potential governance functions of ESG-related frameworks, not as proof of causal effectiveness. In particular, the review supports the proposition that ESG can structure accountability and reporting processes, but it does not demonstrate that ESG adoption alone improves population health outcomes or eliminates governance deficits.

Our findings align with prior analyses of governance frameworks in public health, which emphasize the need for standardized indicators and cross-sectoral collaboration. Several studies in this review [60, 63, 75] confirm that standardized frameworks such as the WHO Europe SDG Roadmap can help governments systematically monitor progress and reinforce accountability. These results mirror the conclusions of broader governance reviews [85, 88] which showed that structured evaluation frameworks support more transparent decision-making and better alignment of health system priorities with the SDGs.

At the organizational level, ESG tools such as disclosure indexes and processing maps [65, 66, 78] were associated with improvements in sustainability reporting and stakeholder engagement. These findings complement earlier corporate responsibility analyses [73, 90] which found that ESG and CSR approaches can create reputational and operational benefits for healthcare organizations. Nevertheless, the variability in adoption and reporting practices across contexts underscores the lack of harmonized standards [69, 82, 91].

Recent studies have examined the impact of ESG frameworks on healthcare organizations’ performance and accountability. Evidence suggests that ESG performance can positively influence financial performance, particularly in developed economies [38, 92]. The environmental pillar shows a concave impact on return on assets, while governance consistently predicts positive financial outcomes [92, 93]. ESG implementation in healthcare can contribute to sustainability goals and offer economic benefits [15, 94]. Local institutional ownership has been found to reduce toxic chemical releases and enhance firm performance through pollution abatement policies [95]. However, the relationship between ESG and performance varies across different contexts and measures [17, 18]. Overall, ESG frameworks appear to have a growing influence on healthcare organizations’ practices and outcomes.

Governance mechanisms in health systems have been shown to influence health outcomes in low- and middle-income countries through decentralization, stakeholder alignment, community engagement, and social capital strengthening [86, 96]. Key governance areas impacting healthcare quality include leadership, system design, accountability, financing, private partnerships, information systems, community participation, and regulation [97, 98]. Good governance enhances the efficacy of health expenditure and service delivery [99]. ESG frameworks can guide sustainable development in healthcare organizations [94], while the SDGs complement ESG standards by focusing on externalities [18]. Effective governance is crucial for translating resources into quality care [100]. In developing countries, non-governmental and international actors play vital roles in connecting global and local health governance [101]. A comprehensive framework for assessing health system governance includes principles such as strategic vision, participation, transparency, and accountability [42].

The evidence reviewed suggests that effective ESG integration depends on multiple enabling conditions. First, strong institutional leadership and political commitment are critical. Bickler et al. [63] and Martins and Paes-Sousa [75] illustrate how leadership can drive alignment between ESG metrics and national reporting obligations, such as Voluntary National Reviews. Second, legal and regulatory frameworks that mandate disclosure, such as the European Corporate Sustainability Reporting Directive (CSRD) analyzed by Zariņš and Siders [83] and Capobianco et al. [68], increase compliance and improve the quality of reporting in public health institutions.

Another implication for practice is the need to strengthen technical capacity for data collection, analysis, and reporting. Several studies [61, 74, 82] emphasized that data gaps and inconsistencies undermine the ability of ESG frameworks to generate actionable insights. Capacity-building efforts must therefore be prioritized, particularly in low-resource settings, as highlighted by Hone et al. [72] and Ahen [90]. Finally, a culture of stakeholder engagement and participatory governance, documented in studies such as Mesa-Vieira et al. [89] and Brizolla et al. [67], is essential to ensure that ESG initiatives are responsive to community needs and foster shared accountability.

A recurring concern across the literature is the risk that ESG remains a reporting exercise rather than a mechanism for substantive institutional change. In public health, this distinction is particularly important because improved disclosure does not necessarily translate into better service quality, equity, or community accountability. Measurement inconsistency, lack of comparable indicators, weak data infrastructures, and fragmented regulatory requirements can generate symbolic compliance or selective reporting, especially when ESG adoption is voluntary or weakly linked to public oversight.

The review also highlights the need to examine ESG integration through the lens of structural inequalities. Low-resource governance environments may lack reliable data systems, technical staff, legal mandates, or financial capacity to implement sophisticated ESG reporting architectures. In these contexts, ESG tools should be adapted to avoid increasing administrative burden or widening inequities between well-resourced institutions and under-resourced public health systems. Practical implementation should prioritize a limited set of feasible, equity-sensitive, and locally validated indicators.

Recent research highlights the growing importance of ESG and sustainability reporting in corporate governance, particularly within the European Union. The Corporate Sustainability Reporting Directive (CSRD) and European Sustainability Reporting Standards (ESRS) are driving significant changes in non-financial disclosure practices [102, 103]. These frameworks aim to enhance transparency, stakeholder trust, and corporate accountability [83, 104]. While implementation challenges persist, including regulatory fragmentation and data reliability concerns [76, 105, 106], studies indicate that effective ESG reporting can improve financial performance and investor confidence [107]. The EU’s proactive approach in mandating sustainability reporting is setting global standards [108], with some countries adopting strict penalties for non-compliance [83]. Future research should focus on refining sector-specific impacts and standardizing reporting practices to ensure comparability and credibility across industries [106].

Successful implementation of ESG indicators in public health institutions requires multifaceted strategies. Key approaches include stakeholder engagement through participatory planning and adaptive program models [109], formation of technical coordination units [110], and clear communication channels [111]. Effective data systems for real-time monitoring and impact assessment are crucial [109], as is aligning efforts with organizational objectives [111]. Multi-sectoral collaboration is essential, involving diverse stakeholders and sectors to leverage combined strengths [112]. This approach can promote transformative change [113] and address complex health challenges [114–116]. Ethical considerations in stakeholder engagement should be guided by a unifying framework integrating core research principles [70]. Successful implementation can lead to improved health outcomes and recognition, as demonstrated by Assuta Medical Centers’ ESG initiatives [94].

This review revealed several strengths in the emerging literature. Many studies used mixed methods allowing for triangulation of findings across qualitative and quantitative data sources. There was also evidence of innovative applications of ESG indicators to public health contexts, including the integration of environmental criteria in health technology assessments and the use of community engagement frameworks in urban governance [111, 112].

However, important limitations persist. ESG integration remains inconsistent across settings, with many studies documenting fragmented adoption and reporting practices. Few studies rigorously evaluated the direct impact of ESG frameworks on health outcomes such as mortality or service quality. The majority of evidence came from high-income or European settings, with fewer contributions from low- and middle-income countries, limiting generalizability.

An additional limitation is that no formal critical appraisal was undertaken, consistent with the scoping review design. Consequently, conclusions about effectiveness are intentionally cautious and framed as hypotheses for future evaluation rather than definitive evidence.

Future research should focus on developing and validating standardized ESG indicators tailored for public health governance. Comparative analyses across different contexts could help identify best practices and contextual factors that facilitate successful implementation. There is also a need for longitudinal studies that measure the impact of ESG frameworks on institutional performance and health outcomes over time.

Moreover, deeper exploration of the social dimensions of ESG is warranted. While several studies addressed equity and participation, these aspects are often underrepresented compared to environmental and governance indicators. Strengthening the evidence base in this area will be critical for ensuring that ESG integration not only improves transparency but also contributes to more inclusive and equitable health systems.

This scoping review highlights the growing relevance of ESG frameworks as instruments for enhancing institutional accountability and sustainability within public health governance. The integration of ESG indicators, when supported by strong leadership, standardized reporting mechanisms, and multisectoral collaboration, can promote transparency, stakeholder engagement, and more effective alignment of health systems with global sustainability goals. However, the evidence reveals significant heterogeneity in implementation, persistent data and reporting gaps, and regulatory inconsistencies that limit the scalability and impact of these approaches across contexts. To address these challenges, efforts should focus on developing and validating sector-specific ESG indicators, harmonizing reporting standards, and strengthening technical and institutional capacity, particularly in low-resource settings. In addition, further research is needed to evaluate the long-term effects of ESG integration on health outcomes and system performance and to better capture the social and equity dimensions of sustainability. Strengthening these foundations will be essential to ensuring that ESG frameworks evolve from voluntary initiatives into effective tools for advancing transparency, accountability, and equitable health governance worldwide.

Overall, the contribution of this review is conceptual and policy-oriented: it clarifies how ESG can be reframed from a corporate disclosure logic into a public governance tool, while showing that this transition requires theoretical precision, validated indicators, and evidence on implementation and impact.
