# Supplementary material for: Integrating ESG indicators into public health governance as a policy innovation for institutional accountability: a scoping review
Source: Public Health Rev. 2026 Aug 17;47:1609720. doi: 10.3389/phrs.2026.1609720 (PMC13526686; doi:10.3389/phrs.2026.1609720)
Supplement: Supplementary file 1 [file Table1.docx]

Supplementary Table S1. Full electronic search strategies for all databases

| **Source** | **Search strategy** |
| --- | --- |
| **PubMed (MEDLINE)** | (("public health governance"[tiab] OR "health system*"[tiab] OR "public health institution*"[tiab] OR "regulatory bod*"[tiab] OR "government agenc*"[tiab]) AND ("environmental social governance"[tiab] OR ESG[tiab] OR "sustainability indicator*"[tiab] OR "sustainable development goal*"[tiab] OR SDG*[tiab] OR "corporate social responsibility"[tiab] OR CSR[tiab]) AND ("institutional accountability"[tiab] OR "policy innovation"[tiab] OR "governance framework*"[tiab] OR "control mechanism*"[tiab])) |
| **Scopus** | TITLE-ABS-KEY("public health governance" OR "health system*" OR "public health institution*" OR "regulatory bod*" OR "government agenc*") AND TITLE-ABS-KEY("environmental social governance" OR ESG OR "sustainability indicator*" OR "sustainable development goal*" OR SDG* OR "corporate social responsibility" OR CSR) AND TITLE-ABS-KEY("institutional accountability" OR "policy innovation" OR "governance framework*" OR "control mechanism*") |
| **Web of Science (WoS)** | TS=("public health governance" OR "health system*" OR "public health institution*" OR "regulatory bod*" OR "government agenc*") AND TS=("environmental social governance" OR ESG OR "sustainability indicator*" OR "sustainable development goal*" OR SDG* OR "corporate social responsibility" OR CSR) AND TS=("institutional accountability" OR "policy innovation" OR "governance framework*" OR "control mechanism*") |
| **Embase** | (('public health governance':ab,ti OR 'health system*':ab,ti OR 'public health institution*':ab,ti OR 'regulatory bod*':ab,ti OR 'government agenc*':ab,ti) AND ('environmental social governance':ab,ti OR ESG:ab,ti OR 'sustainability indicator*':ab,ti OR 'sustainable development goal*':ab,ti OR SDG*:ab,ti OR 'corporate social responsibility':ab,ti OR CSR:ab,ti) AND ('institutional accountability':ab,ti OR 'policy innovation':ab,ti OR 'governance framework*':ab,ti OR 'control mechanism*':ab,ti)) |
| **CINAHL** | ("public health governance" OR "health system*" OR "public health institution*" OR "regulatory bod*" OR "government agenc*") AND ("environmental social governance" OR ESG OR "sustainability indicator*" OR "sustainable development goal*" OR SDG* OR "corporate social responsibility" OR CSR) AND ("institutional accountability" OR "policy innovation" OR "governance framework*" OR "control mechanism*") |
| **WHO Institutional Repository** | Manual search using the keyword combination: *("public health governance" OR "health system*" OR "public health institution*") AND ("ESG" OR "sustainability indicators" OR "SDGs" OR "corporate social responsibility") AND ("institutional accountability" OR "policy innovation")*. Filters: reports and policy documents. |
| **OECD iLibrary** | Advanced search in *all fields* using the same keyword combinations as above; filters applied to *health* and *governance* categories. Reports, working papers, and policy briefs were screened. |
| **World Bank Open Knowledge Repository** | Keyword combinations as above in the main search bar; filters applied to *public health*, *governance*, and *institutional development*. Reports and technical notes were included. |
| **UN SDG Knowledge Platform** | Keyword combinations as above in the main search bar; restricted to SDG 3 (Good Health and Well-being) and SDG 16 (Peace, Justice, and Strong Institutions). Screened event documents, progress reports, and policy briefs. |
| **Google Scholar** | "public health governance" AND (ESG OR "sustainability indicators" OR SDGs OR CSR) AND ("accountability" OR "policy innovation"). Sorted by relevance. The first 200 hits were screened manually by title and abstract. |
